# Supplementary material for: Asymptomatic Plasmodium falciparum carriage at the end of the dry season is associated with subsequent infection and clinical malaria in Eastern Gambia
Source: Malar J. 2024 Jan 17;23:22. doi: 10.1186/s12936-024-04836-y (PMC10792942; doi:10.1186/s12936-024-04836-y)
Supplement: Supplementary file 1 — Additional file 1: Figure S1. varATS and 18S RNA standard curves using 3D7 gDNA. Figure S2. Trend analysis for P. falciparum infection prevalence by age groups in the first cohort, by nested PCR. Figure S3. Parasites prevalence by microscopy according to parasite densities measured by varATS (A) and 18S rRNA qPCR (B) molecular tools. Figure S4. Spatio-temporal P. falciparum prevalence by household in villages J and K. Table S1. Hemoglobin levels (g/dL) and classification of anaemia (WHO, 2011). Table S2. Household-based assessment of P. falciparum infection clusters using Bernoulli spatial scan statistic. [file 12936_2024_4836_MOESM1_ESM.docx]

**Additional Figures and Table**

**Asymptomatic *Plasmodium* *falciparum* Carriage at the End of the Dry Season is Associated with Subsequent Infection and Clinical Malaria in Eastern Gambia**

# Affiliations:

Balotin Fogang**^1^**, Lionel Lellouche**^1^**, Sukai Ceesay**^2^**, Sainabou Drammeh**^2^**, Fatou K Jaiteh**^2^**, Marc-Antoine Guery**^1^**, Jordi Landier**^3^**, Cynthia Haanappel**^4^**, Janeri Froberg**^4^**, David Conway**^5^**, Umberto d’Alessandro**^2^**, Teun Bousema**^4^**, Antoine Claessens**^1,2,4^**

**^1^**LPHI, MIVEGEC, University of Montpellier, CNRS, INSERM, Montpellier, France

**^2^**Medical Research Council Unit The Gambia at the London School of Hygiene and Tropical Medicine, Banjul, The Gambia

**^3^**Aix Marseille Univ, IRD, INSERM, SESSTIM, ISSPAM, 27 boulevard Jean Moulin, 13005, Marseille, France

**^4^**Radboud university medical center, Radboud Institute for Health Sciences, Department of Medical Microbiology, Nijmegen, The Netherlands

^5^Department of Infection Biology, London School of Hygiene and Tropical Medicine, London, UK

Corresponding author: antoine.claessens@umontpellier.fr

# Additional Figures


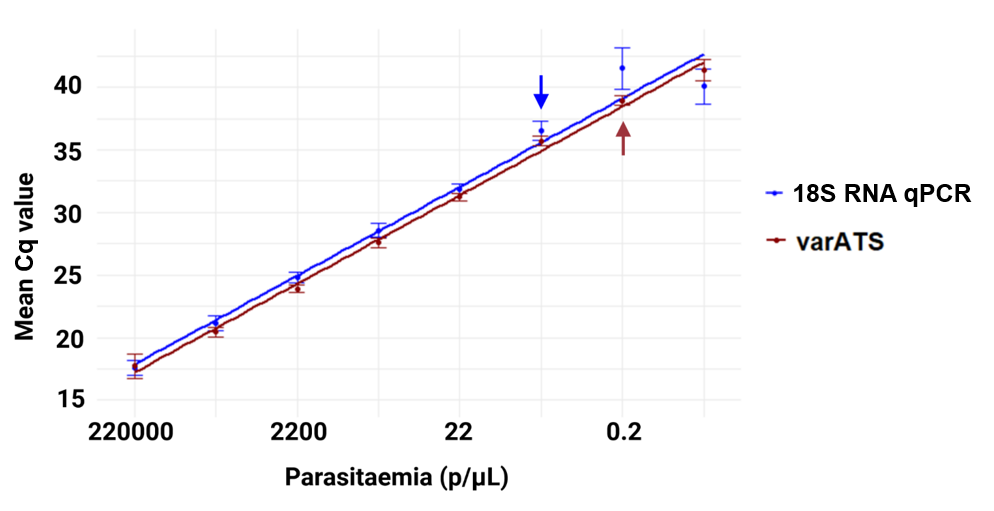


**Figure S1. varATS and 18S RNA standard curves using 3D7 gDNA**. The scatter plot represents the mean Cq value of the amplification of 8 replicates of the 10-fold serial dilution of 220000 parasites/µL of ring stage lab adapted *P. falciparum* 3D7 strain. The arrows indicate the detection limits of the two tests (2.2 parasites/µL for 18S RNA qPCR and 0.2 for varATS). Data were generated with the following method: Ring stage laboratory-adapted *P. falciparum* strain 3D7 was used to determine analytical sensitivity and parasitemia by 10-fold dilution of 220000 parasites/µL of parasitemia in 50% haematocrit from an uninfected blood donor [24]. Dilutions were prepared as a DBS and genomic DNA was extracted as described above. Each DNA sample was tested in duplicate (field isolates and 3D7 strain) and the quantification curve was made from the 3D7 standard dilution. Cut-off values for positivity for 18S rRNA qPCR and varATS were dilutions 6 (parasitaemia of 2.2 parasites/µL) and 7 (parasitaemia of 0.22 parasites/µL), respectively. Samples were defined as *P. falciparum* positive if the Ct value was less than or equal to the Ct of the cut-off. Samples that exhibited amplification for both duplicates and had a visually clear amplification curve were considered positive if their CQ value exceeded dilution 7 but remained below the upper limit of the 95% confidence interval for this parasitemia. Parasite densities were determined using standard curves generated from 3D7 genomic DNA amplifications of all dilutions of each plate with known parasite density.


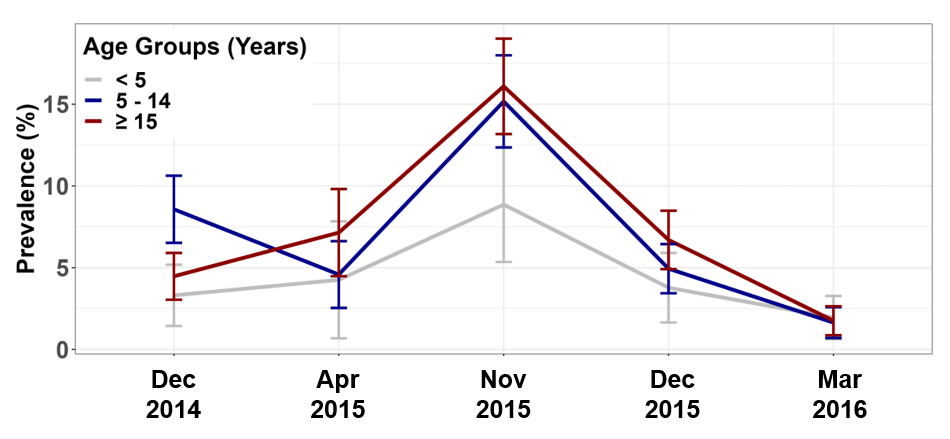


**Figure S2:** **Trend analysis for *P. falciparum* infection prevalence by age groups in the first cohort, by nested PCR.** Error bars represent 95% confidence intervals for prevalence.


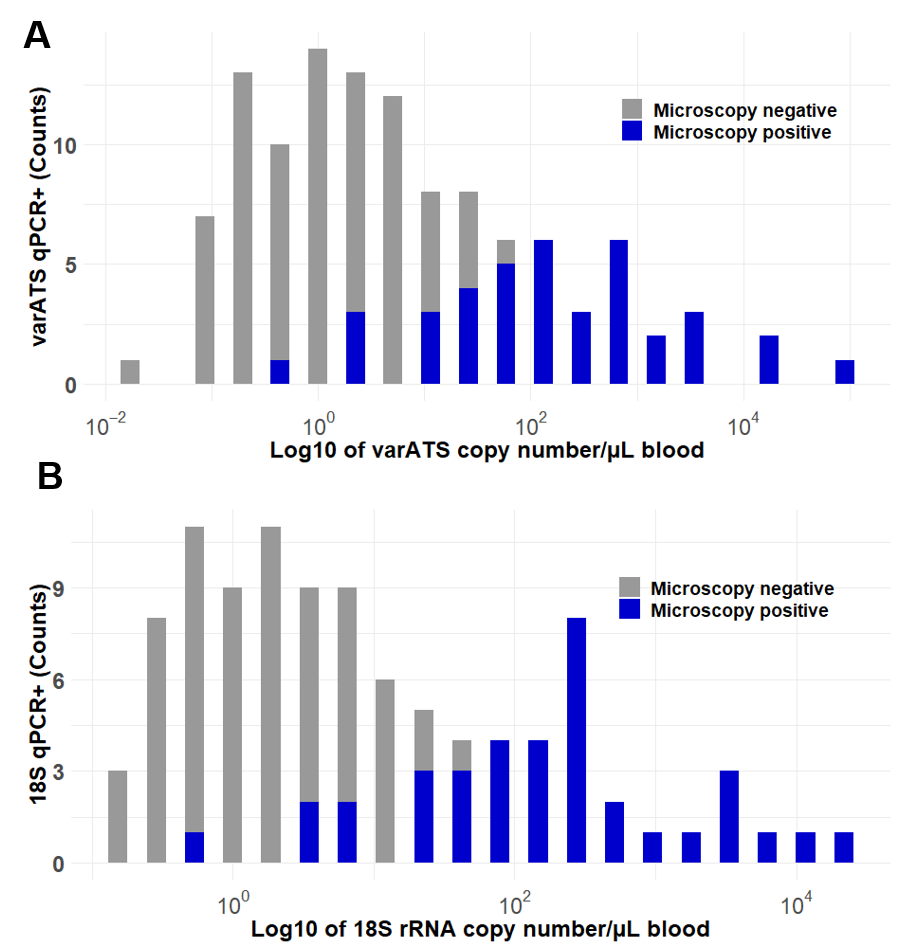


**Figure S3. Parasites prevalence by microscopy according to parasite densities measured by varATS (A) and 18S rRNA qPCR (B) molecular tools.** Parasite densities quantified by varATS and 18S rRNA qPCR are presented (log10 scale with a bin size of 20) for both microscopy negative and microscopy positive.


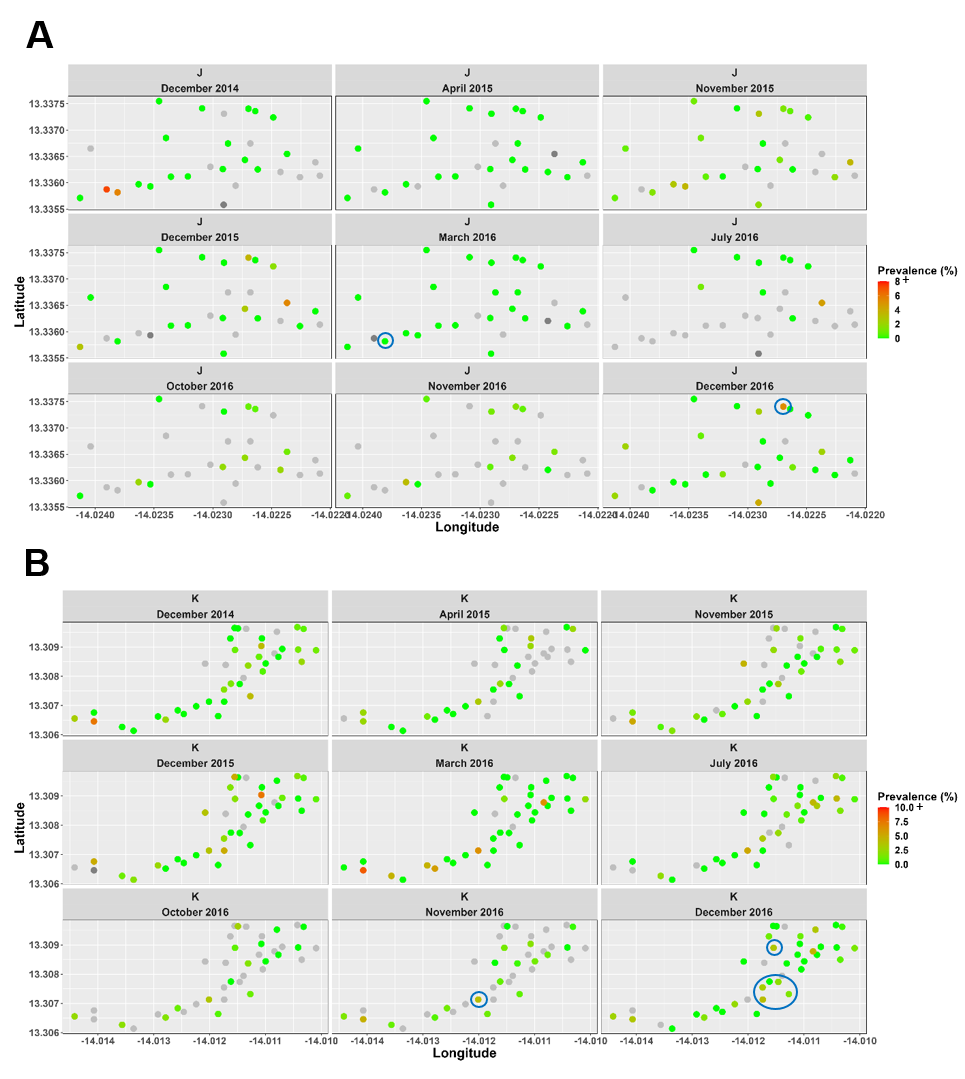


**Figure S4. Spatio-temporal *P. falciparum* prevalence by household in villages J and K.** *P. falciparum* prevalence in each household was normalized to the prevalence of infections per month in each village and only households with at least three individuals were considered. Grey colour represents the household with no data. The blue circles represent the significant hotspots (p< 0.05) determined using the Bernoulli spatial scan statistic in Satscan algorithm.

# Additional Tables

**Table S1.** Hemoglobin levels (g/dl) and classification of anaemia (WHO, 2011)

|  |  | Anaemia status | | |
| --- | --- | --- | --- | --- |
| Population | No anaemia | Mild | Moderate | severe |
| Children 6 to 59 months | ≥ 11.0 | 10.0-10.9 | 7.0-9.9 | < 7 |
| Children 6 to 11 years | ≥ 11.5 | 11.0-11.4 | 8.0-10.9 | < 8 |
| Children 12 to 14 years | ≥ 12.0 | 11.0-11.9 | 8.0-10.9 | < 8 |
| Non-pregnant women > 15 years | ≥ 12.0 | 11.0-11.9 | 8.0-10.9 | < 8 |
| Pregnant women > 15 years | ≥ 11.0 | 10.0-10.9 | 7.0-9.9 | < 7 |
| Men > 15 years | ≥ 13.0 | 11.0-12.9 | 8.0-10.9 | < 8 |

**Table S2: Household-based assessment of *P. falciparum* infection clusters using Bernoulli spatial scan statistic**

| Villages | Sampling  timepoint | Population | Infected | Prevalence of infections (%) | Identified Clusters | P-value |
| --- | --- | --- | --- | --- | --- | --- |
| K | December 2014 | 373 | 30 | 8 | K014 | 0.074 |
|  |  |  |  |  | K001, K003 | 0.126 |
|  |  |  |  |  | K037 | 0.638 |
|  |  |  |  |  | K032 | 0.999 |
|  | April 2015 | 141 | 14 | 9.9 | K021, K022 | 0.53 |
|  |  |  |  |  | K001, K003, K002 | 0.85 |
|  |  |  |  |  | K009 | 0.95 |
|  | November 2015 | 247 | 36 | 14.6 | K003 | 0.231 |
|  |  |  |  |  | K036 | 0.366 |
|  |  |  |  |  | K013, K014 | 0.954 |
|  | December 2015 | 375 | 25 | 6.2 | K002, K003 | 0.107 |
|  |  |  |  |  | K021 | 0.478 |
|  |  |  |  |  | K020 | 0.794 |
|  |  |  |  |  | K038, K009, K010 | 0.863 |
|  | March 2016 | 460 | 9 | 2 | K042, K005, K006, K041, K003 | 0.289 |
|  | July 2016 | 366 | 28 | 7.7 | K001, K003 | 0.255 |
|  |  |  |  |  | K028, K030, K027, K031 | 0.444 |
|  |  |  |  |  | K010, K011, K036, K038, K009 | 0.622 |
|  |  |  |  |  | K024 | 0.995 |
|  | October 2016 | 226 | 16 | 7.1 | K001, K003, K005, K041, K007, K009 | 0.173 |
|  |  |  |  |  | K019 | 0.91 |
|  |  |  |  |  | K015 | 0.997 |
|  | November 2016 | 185 | 39 | 21.1 | **K009** | **0.031** |
|  |  |  |  |  | K003 | 0.065 |
|  |  |  |  |  | K017, K021 | 0.971 |
|  | December 2016 | 449 | 64 | 14.2 | **K017** | **0.0025** |
|  |  |  |  |  | **K037, K036, K038, K010** | **0.0036** |
|  |  |  |  |  | K001, K003 | 0.334 |
|  |  |  |  |  | K023 | 0.712 |
|  |  |  |  |  | K030 | 0.776 |
|  |  |  |  |  | K026 | 0.985 |
|  |  |  |  |  | K008 | 0.999 |
| J | December 2014 | 201 | 3 | 1.5 | J014, J016 | 0.382 |
|  | April 2015 | 147 | 1 | 0.7 | NC |  |
|  | November 2015 | 184 | 26 | 14.1 | J013, J017 | 0.342 |
|  |  |  |  |  | J003, J006, J004 | 0.664 |
|  |  |  |  |  | J023 | 0.938 |
|  | December 2015 | 12 | 6 | 50 | NC |  |
|  | March 2016 | 226 | 3 | 1.3 | **J016** | **0.023** |
|  | July 2016 | 227 | 4 | 3.1 | J005, J006, J011, J009, J012, J022, J004, J018 | 0.357 |
|  | October 2016 | 82 | 10 | 12.2 | J007, J009 | 0.66 |
|  |  |  |  |  | J005, J011 | 0.72 |
|  | November 2016 | 101 | 23 | 22.8 | J017 | 0.265 |
|  |  |  |  |  | J005 | 0.963 |
|  |  |  |  |  | J027 | 0.974 |
|  | December 2016 | 232 | 19 | 8.2 | **J027** | **0.0054** |
|  |  |  |  |  | J022 | 0.841 |
|  |  |  |  |  | J003 | 0.996 |
|  |  |  |  |  | J028 | 0.996 |
|  |  |  |  |  | J015 | 0.999 |

*P. falciparum* infections clusters were identified using Bernoulli spatial scan analysis method in SaTScan version 10.1.2. NC: no cluster identified. In bold, the group with a significantly higher prevalence of *Plasmodium* infection (p< 0.05) compared to other households at a given time.
